# USING TRANSFER LEARNING TO IMPROVE ALGORITHMIC DEMENTIA CLASSIFICATION ACROSS DIFFERENT RACIAL AND ETHNIC GROUPS

**DOI:** 10.1093/geroni/igae098.4347

**Published:** 2024-12-31

**Authors:** Anja Leist, M Maria Glymour, Kenneth Langa, Jung Hyun Kim

**Affiliations:** University of Luxembourg, Esch-sur-Alzette, Grevenmacher, Luxembourg; Boston University, Boston, Massachusetts, United States; University of Michigan, Ann Arbor, Michigan, United States; University of Luxembourg, Esch-sur-Alzette, Grevenmacher, Luxembourg

## Abstract

**Background:**

In the absence of clinical assessments, algorithmic ‘probable dementia’ estimation aids public health and epidemiological research on determinants and consequences of dementia. Algorithmic performances have differed by social and racial/ethnic groups due to heterogeneous sample sizes. In this study, we present improvements in the accuracy of group-specific ‘probable dementia’ estimation using a transfer learning approach. Transfer learning – a novel approach not previously applied in algorithmic dementia classifications – uses a large “source” dataset with imprecise (non-clinical) outcome assessments to develop an initial algorithm with a large and diverse sample, complemented by a smaller “target” dataset with high-quality (clinical) outcome assessments to develop models with refined estimation of model coefficients.

**Methods:**

Data came from the Health and Retirement Study (source data: N=6,630) and the Harmonized Cognitive Assessment Protocol (target data: N=2,388). Different algorithms to estimate probable dementia were evaluated through overall accuracy (Brier score), calibration (intercept, slope), and discriminative ability (area under the receiver operating characteristic curve, AUC; area under the precision-recall curve, AUPRC).

**Results:**

Among non-Hispanic Black participants, the transfer-learned algorithm had higher accuracy than the best previously reported algorithm (Brier 0.049 vs. 0.061; AUC 0.84 vs. 0.81; AUPRC 0.52 vs. 0.39). Among Hispanic participants, the transfer-learned algorithm improved model calibration (Intercept −0.07 vs. −1.19; Slope 0.88 vs. 0.08).

**Discussion:**

The use of transfer learning can improve algorithms to estimate probable dementia from non-clinical assessments for groups historically underrepresented in research. More accurate classification will lead to more equitable epidemiological and public health research on dementia.

